# An effective and robust method for tracking multiple fish in video image based on fish head detection

**DOI:** 10.1186/s12859-016-1138-y

**Published:** 2016-06-23

**Authors:** Zhi-Ming Qian, Shuo Hong Wang, Xi En Cheng, Yan Qiu Chen

**Affiliations:** School of Computer Science, Shanghai Key Laboratory of Intelligent Information Processing, Fudan University, Shanghai, China; Chuxiong Normal University, Chuxiong, China

**Keywords:** Fish detection, Fish tracking, Global optimization, Occlusion

## Abstract

**Background:**

Fish tracking is an important step for video based analysis of fish behavior. Due to severe body deformation and mutual occlusion of multiple swimming fish, accurate and robust fish tracking from video image sequence is a highly challenging problem. The current tracking methods based on motion information are not accurate and robust enough to track the waving body and handle occlusion. In order to better overcome these problems, we propose a multiple fish tracking method based on fish head detection.

**Results:**

The shape and gray scale characteristics of the fish image are employed to locate the fish head position. For each detected fish head, we utilize the gray distribution of the head region to estimate the fish head direction. Both the position and direction information from fish detection are then combined to build a cost function of fish swimming. Based on the cost function, global optimization method can be applied to associate the target between consecutive frames. Results show that our method can accurately detect the position and direction information of fish head, and has a good tracking performance for dozens of fish.

**Conclusion:**

The proposed method can successfully obtain the motion trajectories for dozens of fish so as to provide more precise data to accommodate systematic analysis of fish behavior.

**Electronic supplementary material:**

The online version of this article (doi:10.1186/s12859-016-1138-y) contains supplementary material, which is available to authorized users.

## Background

Video based fish behavior analysis has become a hot research topic thanks to recent advances in computer vision methods [1–6]. To achieve such goal, it is necessary to first obtain the trajectory data for each fish by tracking their waving bodies, and then perform various statistical computing to discover interesting motion patterns and underlying rules. The robustness and accuracy of the tracking system can directly influence the effectiveness of behavior analysis. Therefore, fish tracking is the key step in the analysis of fish behavior. Because the fish body is not rigid, its shape changes during swimming; in addition, fish often mutually occlude, which has brought great difficulties for the fish tracking in video image.

Existing fish tracking methods are mainly based on motion information [7–10]. It predicts the position of the fish at the next moment by analyzing the motion state of each detected fish. Such method can track a large number of fish at the same time, but the tracking accuracy and stability are not good. In order to solve this problem, Pérez-Escudero et al. [11] put forward a tracking method based on appearance information. They conduct an appearance analysis for each detected fish to obtain the feature of “fish fingerprints”, and then associate with the targets that have the same “fish fingerprints” in different frames to obtain their motion trajectories. This method can correctly identify each individual even after crossings or occlusions, and can be applied to track different kinds of animals; but when the number of tracked objects is large, the identification error may occur due to the similarity of appearance between objects. Therefore, it is not suitable for tracking a large number of fish. There are usually lots of targets in the analysis of group behavior, so it is better to choose the tracking method based on motion information.

Detection error, motion prediction and mutual occlusion are the three most challenging problems for tracking methods based on motion information. Detection error usually includes two categories: missing detection and error detection, and they can directly affect the accuracy of follow-up tracking. The missing detection is inevitable because there is occlusion among fish in the process of their movements. In order to improve the tracking performance, the error detection rate must be minimized. Furthermore, due to the randomness of fish movements, it is difficult to analyze all of their motion state accurately by using a single motion model, and if a mixture motion model is used, though it can improve the tracking performance to some degree, meanwhile it also increases the tracking difficulty and complexity, which is not conducive to the realization of tracking. Finally, the mutual occlusion of fish can result in missing detection, and the longer the occlusion time is, the longer the missing detection time will be, which can cause the fragmentation of motion trajectory and thus degrade the tracking performance.

Our observation indicates that although fish movements are random, there is a good motion consistency to maintain the continuity of position and direction of the same target between consecutive frames. As long as the error detection rate in the detection phase can be decreased and meanwhile the direction of fish movements can be gotten, the targets between consecutive frames can be associated according to the position and direction information even without the use of motion prediction. Based on the above analysis, we put forward a multiple fish tracking method based on fish head detection. It has the following characteristics: (1) It can simultaneously detect the position and direction information of fish head and has a low error detection rate; (2) Without the use of motion model to conduct the motion prediction for fish, it greatly simplifies tracking processes; (3) It can better solve the occlusion problem in fish swimming and improves the tracking stability. The experimental results show that it can conduct a motion tracking for dozens of fish and has a good tracking performance.

## Methods

**Fig. 1:**
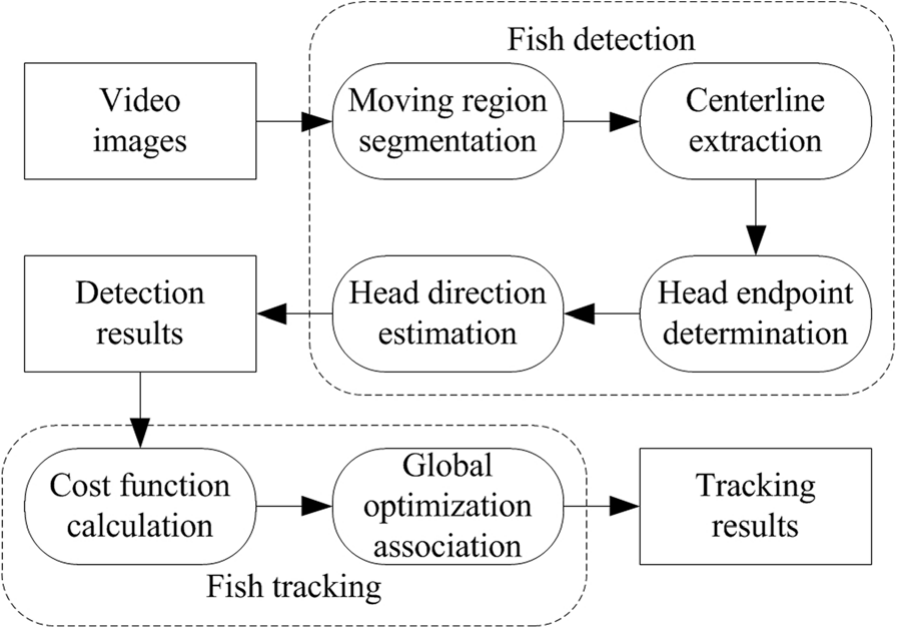
The flow chart of the proposed method. It is comprised of two stages: fish detection and fish tracking

The proposed method is comprised of two stages: fish detection and fish tracking. During the detection phase, the centerline of the moving region in video image is first extracted; then the fish head position is found based on the endpoint width of the centerline, and finally the fish head direction is estimated according to the gray distribution around the endpoint; During the tracking phase, according to the position and direction information of the detected fish head, the fish head between consecutive frames is associated through the use of global optimization method to obtain their motion trajectories. The whole process is shown in Fig. 1, each of the steps in the figure are described sequentially as follows.

### Fish detection

**Fig. 2:**
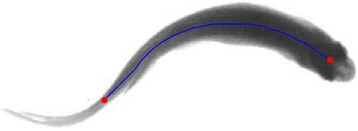
An illustration of the centerline of the fish body. The centerline (blue line) represents the main structure of fish, and the endpoints (red points) of the centerline are located at fish head and tail

As shown in Fig. 2, a fish appears as an elongated body in a top view image. Its centerline is a good approximation to its geometry, which greatly reduces complexity by decreasing a 2D region to a 1D curve while keeping its main shape characteristics and consistent with its spinal structure. Along the centerline, the head region is wider than that of the tail region, and the head and tail are on the end positions of the centerline. During swimming, the fish head is the part which experiences least shape change, the direction of the head can better indicate the forward direction of the fish and since the head region is only a small part in the fish body, it is affected by the occlusion to the smaller probability. Following the above cues, the proposed method is designed to consist of the following steps to detect the fish head.

### Moving region segmentation

For laboratory study of fish behavior, the captured video contains only swimming fish and nearly static background, and most of the moving targets stay only for a short time in an area, it is possible to detect the moving region by modeling background with the time domain-based median filtering method [12]. First, the median images of the first *n* frames in the video can be selected as a background image, and then the moving regions can be segmented by setting a threshold for the differential image of the background image and the input image.

1 $$ {R}_t=\left\{\left(x,y\right)\ \in I\left|\kern1em \right|\underset{\left({I}_1,\dots, {I}_n\right)}{median}\left(x,y\right)-{I}_t\left(x,y\right)\Big|,>,{T}_g\right\} $$

where *I*_*t*_(*x,y*) denotes the *t*-th frame image, and *R*_*t*_(*x,y*) represents the obtained moving regions. In order to facilitate subsequent centerline extraction, the obtained moving regions are first filled to eliminate the holes. Next, the small interference blocks are filtered out; finally, the regions are smoothed through median filtering.

### Centerline extraction

Centerline extraction procedures will be performed on moving regions obtained in the previous step. There are a number of existing methods for extracting the centerline of a region. In order to efficiently describe belt-like fish body, the augmented fast marching method (AFMM) [13] is adopted to extract the centerline. The basic idea of the AFMM is to construct an active narrowband in the peripheral image region. The arrival time *U* of the internal points of the active narrowband is undefined; the current spreading boundary transmits inward by using a reverse difference scheme and as the point spread, they are frozen at arrival time *U*, and then construct a new moving narrowband.

The centerline can be regarded as points that collapse when the edge of regions is propagated in the AFMM. Each propagated point has a source point at the edge. Hence, centerline of region can be extracted by locating points at edge corresponding to each of the propagated points.

First, we randomly choose a point at the edge of the region and let the arrival time of this point be *U* = 1. We start with this point, continuously increasing *U* to initialize *U* of all edge points. Later, the *U* values of all points throughout the region are determined using the AFMM, and the *U* value of each point is consistent with that of the nearest edge point. Thus, the centerline *C* can be obtained using the following equation:

2 $$ \begin{array}{l}C=\left\{\left(i,j\right)\Big| \max \left(\left|ux\left|,\right|uy\right|\right)>2\right\}\\ {}ux=U\left(i+1,j\right)-U\left(i,j\right),\kern1em uy=U\left(i,j+1\right)-U\left(i,j\right)\end{array} $$

The above equation represents that the point belongs to the centerline when the maximum difference of the arrival time *U* between this point and two points in the *x* and *y* directions is greater than 2. The AFMM is fast and robust. But due to the complex shape of the moving region, the regional centerline obtained by the AFMM may carry burrs. In order to remove the effects of burrs on the subsequent analysis, a threshold is set to eliminate small branches on the centerline. The final centerline *C* is defined as:

3 $$ C=\left\{\left(i,j\right)\Big| \max \left(\left|ux\left|,\right|uy\right|\right)>{T}_u\right\} $$

**Fig. 3:**
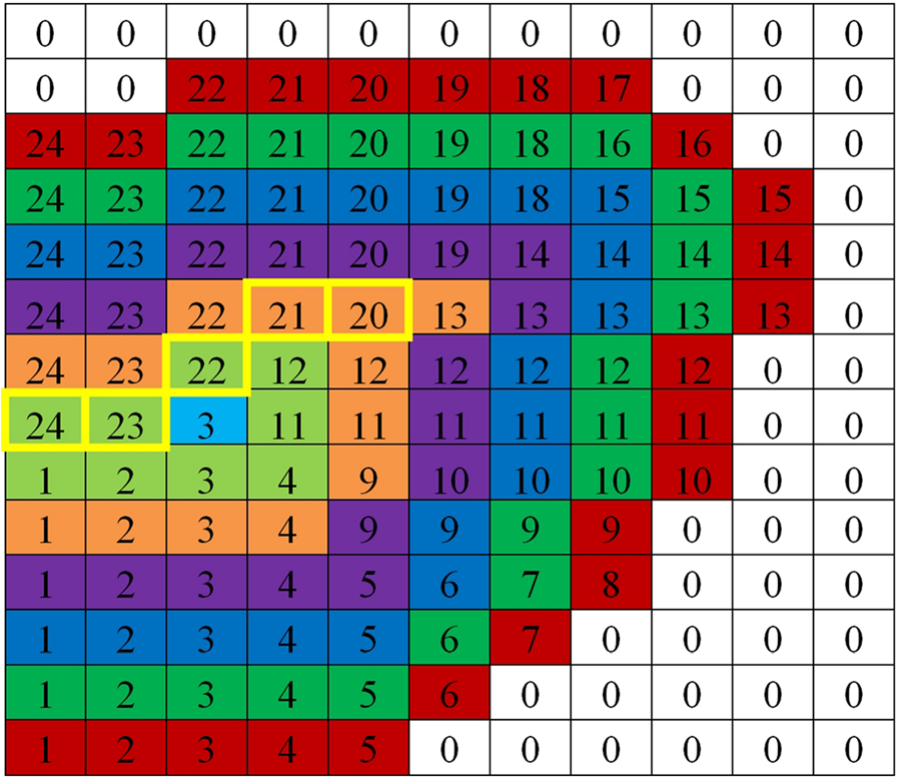
An illustration of the centerline is obtained through the AFMM. Each color represents a transmission process of the value of *U* and the yellow cell indicates the obtained regional centerline when *T*_*u*_ = 7

The threshold *T*_*u*_ in the equation above implies how far the region is observed. A small value for *T*_*u*_ means the region is observed at a short distance, the centerline has more details, and more branches are preserved. On the contrary, a large value for *T*_*u*_ means the region is observed at a long distance, the centerline has few details, and few branches are preserved. Figure 3 shows an example of the arrival time *U* of the fish head region.

**Fig. 4:**
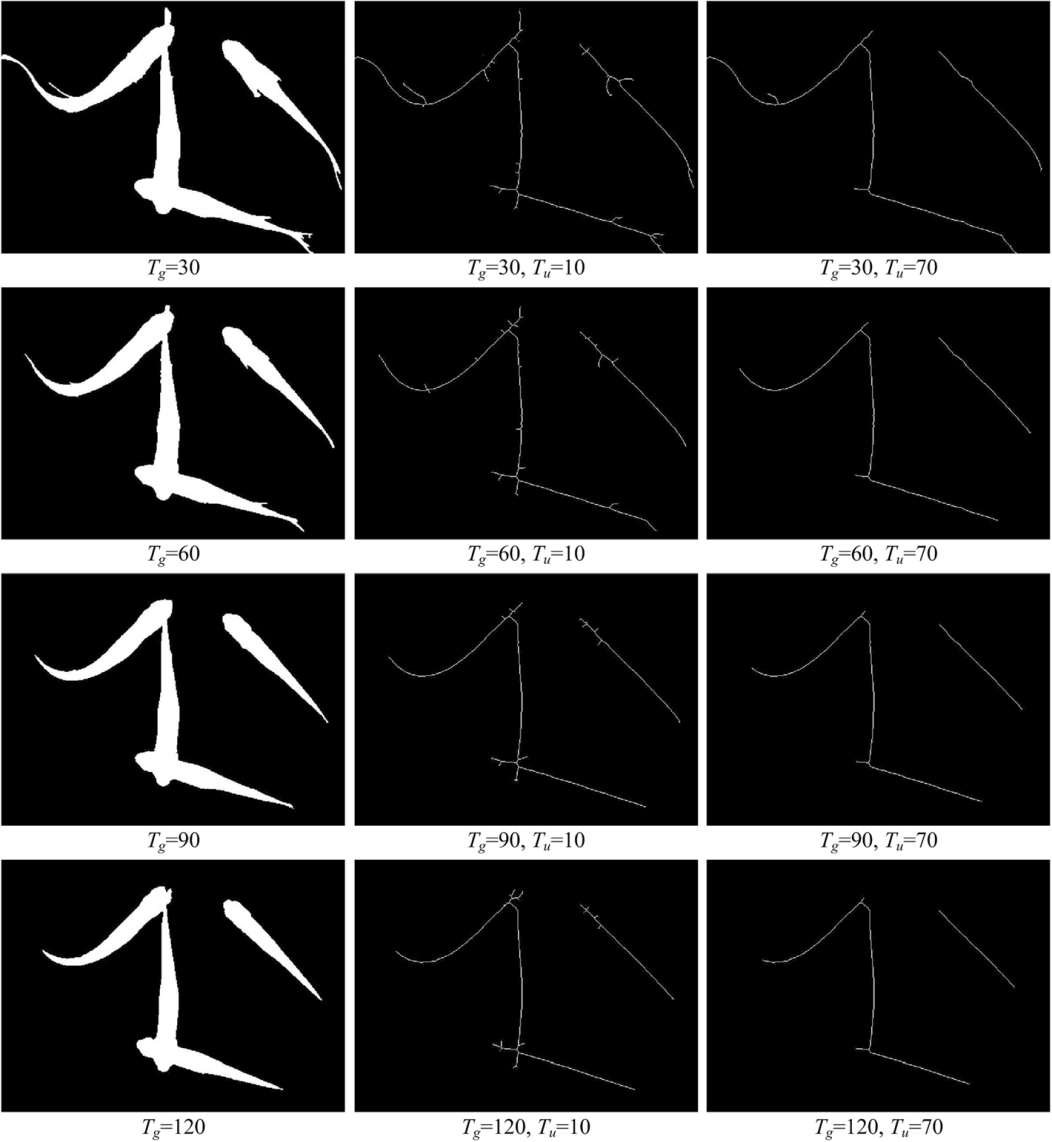
The extracted centerlines versus segmentation results under varying *T*_*g*_ values

Moving region segmentation result influences the extraction of centerline. A small threshold *T*_*u*_ means that the segmentation threshold *T*_*g*_ has more influence on centerline extraction, and a large threshold *T*_*u*_ means that the segmentation threshold *T*_*g*_ has less influence. Figure 4 shows extracted centerlines versus segmentation results under varying *T*_*g*_ values. It is clear from this figure that the influence of moving region segmentation results on centerlines decreases with an increase in threshold *T*_*u*_.

**Fig. 5:**
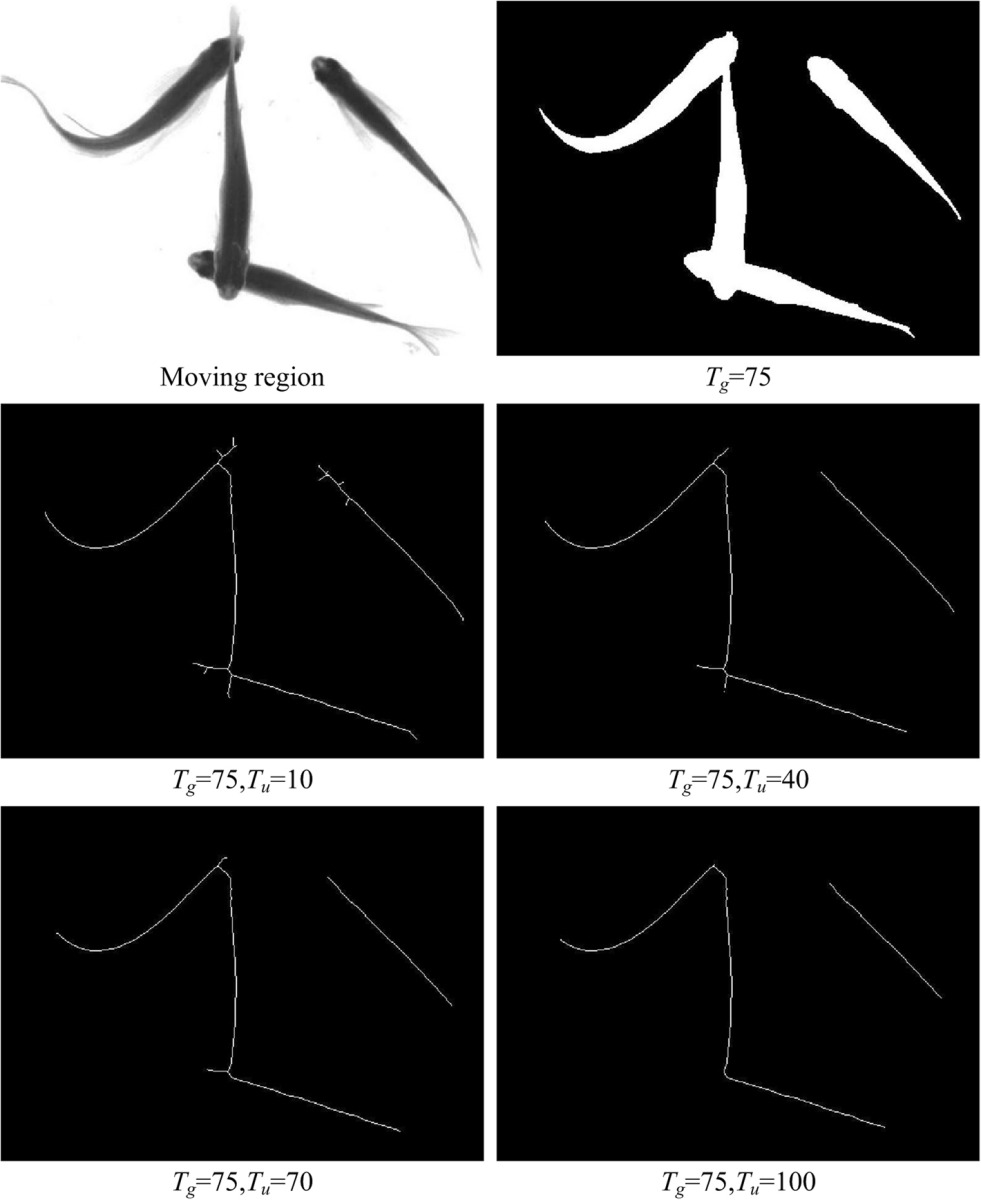
The obtained centerlines in the moving regions with different *T*_*u*_. As *T*_*u*_ increases the centerline can better describe the main regional structure while worse describe the details

When fish occlude, head endpoints may occur at the branch of centerline. A small value of threshold *T*_*u*_ means the head branch is likely to be kept. A large value of *T*_*u*_ means the head branch is likely to be removed. Although a small threshold helps keep more head braches, it is more challenging to analyze the centerline and it decreases the detection performance. Hence, we set a large threshold for extraction of the centerline and ignore details in small branches of the moving region while maintaining the centerline of the fish body’s curved structure. By doing so, we may lose some head branches, but the error detection rate is reduced and tracking performance is improved. Figure 5 shows the extracted centerlines from the same image with different thresholds. It can be seen from this figure that while *T*_*u*_ > =40, the main structure of the fish body centerline is preserved and the fine burrs is ignored.

When tracking occlusions, if head branches are removed, the head cannot be detected in centerline. In this case, based on the association rule (see “Global optimization association” section), tracking of this head is halted and the state of the head from the previous frame is maintained. Head branches will grow larger in subsequent frames when the fish moves. The head will continue to be tracked when the head branch occurs in centerline.

### Head endpoint determination

Centerline characterizes the primary shape structure of a top view fish image and the endpoints of the curve indicate the positions of the fish head and tail. No matter how the centerline changes its shape, the obtained endpoints are usually located in the region of fish head or tail. However, because occlusions will cause complex shape variations during movement of fish, in rare cases, not only the head and tail of the fish, but other body parts may also have branch endpoints in centerlines. In order to remove these endpoints, we set a length threshold *T*_*l*_ to filter all endpoints. When the distance between the endpoint and nearest intersection point is greater than *T*_*l*_, the endpoint will be regarded as the endpoint of the fish head or tail. Otherwise, this endpoint should be removed.

**Fig. 6:**
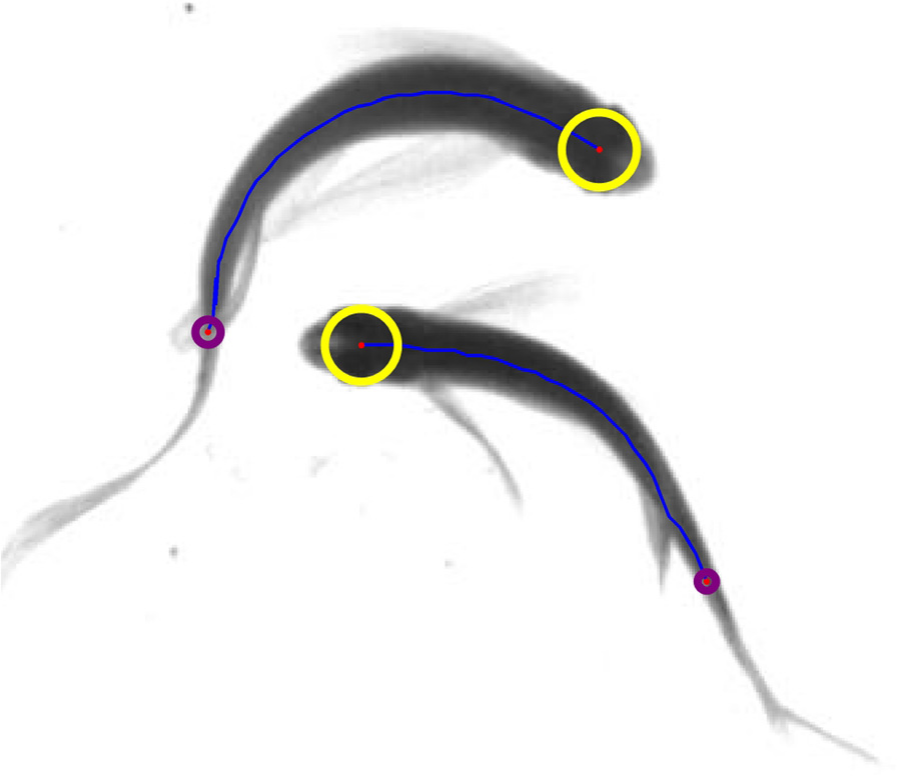
An illustration of the endpoint width of the centerlines. The yellow circles represent the width of fish head endpoints while the purple circles represent the width of fish tail endpoints. Since the fish head width is bigger than fish tail width, the head endpoints can be determined effectively through threshold

Since the width of fish head is larger than that of tail, the endpoint is taken as the circle center and the minimum distance from this point to the edge of the moving region is taken as the semi-diameter of the circle; then the diameter can indicate the region width of the endpoint position approximately, and then whether the endpoint is the fish head endpoint through the set width threshold *T*_*w*_. Figure 6 shows the width comparison results of the head and tail endpoint. Since the deformation of fish head region is little in the process of moving, the obtained endpoint of the fish head is comparatively stable. This ensures the accuracy of the same target position between different image frames.

### Head direction estimation

The previous step obtains the endpoint of the fish head which provides position information. In order to better analyze fish’s motion behavior, we estimate fish head direction by performing multiscale analysis of the Hessian matrix.

The Hessian matrix of the image function describes local structural information. Its eigenvalues and eigenvectors can be used to indicate the curvature and direction in the regional orthogonal direction [14, 15]. With this characteristic, the Hessian matrix of the head endpoint is used to estimate the direction of the head region.

Suppose the head endpoint is (*x*,*y*), the Hessian matrix of the point is defined as:

4 $$ H\left(x,y,s\right)=\left[\begin{array}{l}{L}_{xx}\kern1em {L}_{xy}\\ {}{L}_{xy}\kern1em {L}_{yy}\end{array}\right] $$

where *L*_*xx*_, *L*_*xy*_ and *L*_*yy*_ are the convolution results of the second order Gaussian derivatives with the input image at point (*x*,*y*) at scale *s*. The Determinant of Hessian (DoH) of matrix *H* can be expressed as:

5 $$ D\left(x,y,s\right)=\left({L}_{xx}\times {L}_{yy}-{L_{xy}}^2\right)\times {s}^4 $$

The different DoH values can be obtained at different scales *s*. The scale *ŝ* that generates the maximum DoH value can be obtained as following:

6 $$ \left(x,y,\widehat{s}\right)=\mathrm{argmax}\left|D\left(x,y,s\right)\right| $$

**Fig. 7:**
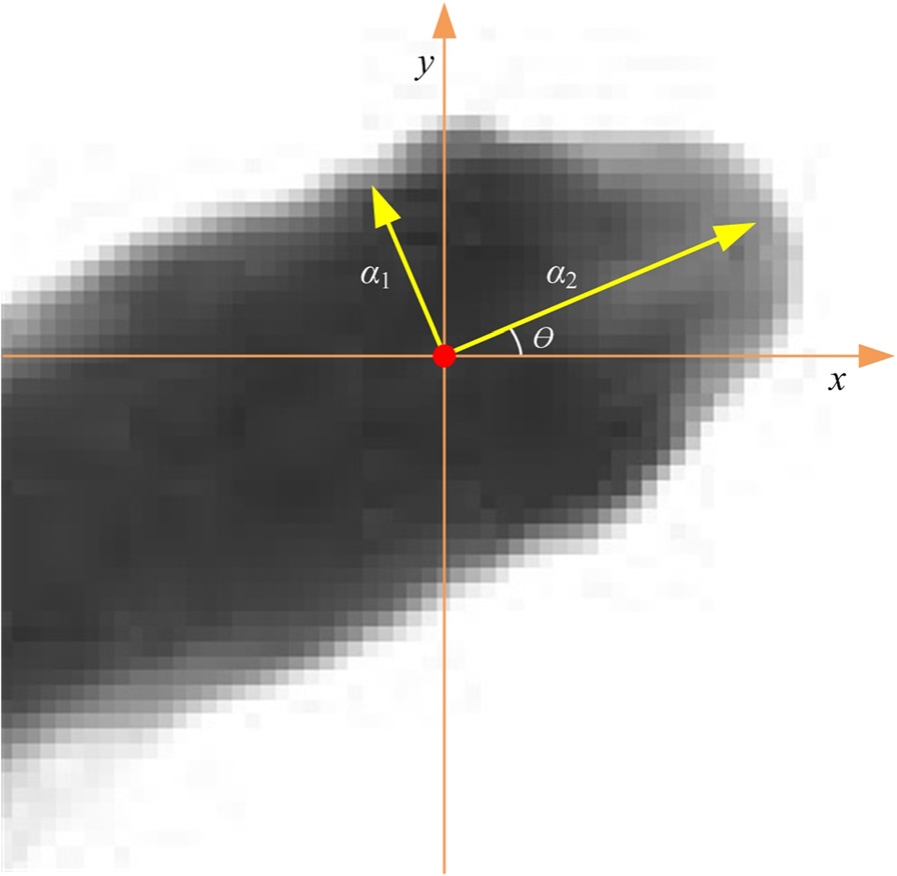
An illustration of the estimation of the head direction. The red point represents the head endpoint position, *α*_1_ and *α*_2_ represent the directions of maximum and minimum curvature at the head endpoint respectively while *θ* represents the angle of the eigenvector *α*_2_ (head direction)

The final Hessian matrix can be expressed as *H*(*x*, *y*, *ŝ*). Let *λ*_1_ and *λ*_2_ (|*λ*_1_| > = |*λ*_2_|) be eigenvalues of the Hessian matrix, *α*_1_ = (*α*_11_,*α*_12_)^*T*^ and *α*_2_ = (*α*_21_,*α*_22_)^*T*^ be the corresponding eigenvectors, respectively. Then, *α*_1_ denotes the direction of maximum curvature at the endpoint of the fish head, and *α*_2_ denotes the direction vertical to the direction of maximum curvature, as shown in Fig. 7. As can be seen from the figure, the direction of *α*_2_ is almost consistent with the direction of the fish head. The first and second dimensions of the eigenvector relate to the *x* and *y* coordinates, respectively. Hence, the fish head direction, i.e., the angle of the eigenvector *α*_2_, can be expressed as *θ* = arctan(*α*_22_/*α*_21_).

### Fish tracking

After the detection of fish head, it becomes possible to track the fish based on the detected information. However due to the randomness and the frequent occlusion when fish swims, it’s very hard to obtain accurately the motion trajectory of fish based on the common motion prediction method that has been used in the multi-target tracking. In order to solve this problem, we propose a target association method according to the continuity of motion information between consecutive frames. First we construct a cost function of fish swimming according to the position and direction information of fish head. Then the cost function values are calculated based on the cost function for different targets between consecutive frames. Finally based on the global optimization of cost function value, the targets in the consecutive frames can be associated directly to get their motion trajectory.

### Cost function calculation

Although it is impossible to accurately model fish motion, we observe that there are some relationships between the motion state of fish head and the position and direction of fish head between consecutive frames. This relationship mainly embodies as follows: between consecutive frames, there is a smaller change in the position and direction of fish head for the same target, while there is a bigger change in the position and direction of fish head for the different target. In order to use this rule in the process of tracking and meanwhile simplify calculations, we define the cost function of the *i*-th target in the previous frame and the *j*-th target in the current frame as follows:

7 $$ c{v}_{ij}=\omega \left(\frac{p{c}_{ij}}{p{c}_{\max }}\right)+\left(1-\omega \right)\left(\frac{d{c}_{ij}}{d{c}_{\max }}\right) $$

where, *pc*_max_ and *dc*_max_ denote the maximum change of position and the maximum change of direction between consecutive frames, respectively; *pc*_*ij*_ and *dc*_*ij*_ denote the change of position and the change of direction between the target *i* in the previous frame and the target *j* in the current frame, respectively; *ω* and (1-*ω*) denote the weight of position change rate and direction change rate in the cost function, respectively.

### Global optimization association

With the cost function, the targets between consecutive frames can be associated through the use of global optimization method. Assuming the number of target in the previous frame is *n* and the number of target in the current frame is *m*, a cost function matrix can be expressed as:

8 $$ A=\left[\begin{array}{l}c{v}_{11}\kern1em c{v}_{12}\kern1em \cdots \kern1em c{v}_{1m}\\ {}c{v}_{21}\kern1em c{v}_{22}\kern1.6em \cdots \kern1em c{v}_{2m}\\ {}\kern1.2em \vdots \kern2.6em \vdots \kern1.94em \vdots \kern2.1em \vdots \\ {}c{v}_{n1}\kern1em c{v}_{n2}\kern1.2em \cdots \kern1em c{v}_{nm}\end{array}\right] $$

In order to reduce the number of association and improve the tracking performance, we set the cost function matrix as follows:

9 $$ c{v}_{ij}=\left\{\begin{array}{l}c{v}_{ij},\kern1em  if\kern0.5em  distance\left(i,j\right)<{T}_o\\ {}+\infty, \kern1em  otherwise\end{array}\right.\kern1em \left(i=1,\dots, n;\kern0.5em j=1,\dots, m\right) $$

where *T*_*o*_ denotes the maximum occlusion distance, *distance*(*i*,*j*) denotes the distance change between the target *i* and the target *j*. The above equation represents that only when the distance change between targets between consecutive frames is less than the maximum occlusion distance can the target be associated; otherwise, the target is not associated.

The optimal association model can be expressed as:

10 $$ \begin{array}{l}Z= \min {\displaystyle \sum_{i=1}^n{\displaystyle \sum_{j=1}^mc{v}_{ij}}}{x}_{ij}\\ {}s.\kern0.5em t.\left\{\begin{array}{l}{\displaystyle \sum_{i=1}^n{x}_{ij}=1\kern1em \left(j=1,\dots, m\right)}\\ {}{\displaystyle \sum_{j=1}^m{x}_{ij}=1\kern1em \left(i=1,\dots, n\right)}\\ {}{x}_{ij}=1\kern0.5em \mathrm{or}\kern0.5em 0\kern1em \left(i=1,\dots, n;\kern0.5em j=1,\dots, m\right)\end{array}\right.\end{array} $$

where *x*_*ij*_ = 1 means the target *i* is associated with the target *j*; *x*_*ij*_ =0 means the target *i* is not associated with the target *j*.

The Hungary algorithm [16] is used to solve the equation above. Due to occlusion among targets, three cases may occur for *n* and *m* during the association. Details of these cases and corresponding processing are given below:

1. *m* = *n*: Two consecutive frames have the same number of targets and are fully matched.
2. *m* > *n*: The current frame has more targets than the previous frame. This implies that new targets occurred, resulting in more associated targets. In this case, among the *m* targets in the current frame, *n* targets are chosen based on Equation (10) to associate with the previous frame. The remaining *m*-*n* targets are ignored to ensure the number of associated targets in the current frame equals *n*.
3. *m* < *n*: The current frame has fewer targets than the previous frame. This means that tracked targets disappeared, resulting in fewer associated targets. In this case, all targets of the current frame are first associated with the previous frame based on Equation (10). As for the non-associated *n*-*m* targets in the previous frame, we maintain the state of these targets from the previous frame for the current frame to ensure the number of associated targets in the current frame equals *n*.

The above steps can be followed to guarantee that two consecutive frames have the same number of associated targets. In the tracking process, consider that the number of targets *N* stays constant, if the number of targets in the initial frame is equal to *N*, the number of associated targets for all subsequent frames remains the same. This method solves the problem of trajectory breaks that occur due to track merging and splitting when fish occlude.

## Results and discussion

### Test sets

In order to evaluate the proposed method, we choose zebrafish (*Danio rerio*) with different densities in two groups as the test data.

- D1: 20 zebrafish are put in strong indoor lighting condition and the tank size is 20 cm × 20 cm filled with water of 3 cm deep. The video frame rate is 40 frames per second.
- D2: 40 zebrafish are put in ordinary indoor lighting condition and the tank size is 30 cm × 30 cm filled with water of 3 cm deep. The video frame rate is 30 frames per second.

The shooting equipment is Flare 4 M180-CL high-speed camera with the image resolution being 2048 × 2040 pixels. All videos are top-view shooting with the length of zebrafish being 1.5-3 cm. From each group of video sequence, 2000 frames of images in which fish behaviors are active and the phenomenon of occlusion exists, are selected as the final test sets.

### Evaluation criteria

The average correct detection rate (ACDR), average error detection rate (AEDR), average occlusion detection rate (AODR) and average direction error (ADE) are used as detection evaluation criteria, and their definitions are as follows: (1) Average correct detection rate: a ratio of the total number of correctly detected targets to the total number of targets; (2) Average error detection rate: a ratio of the total number of wrongly detected targets to the total number of targets; (3) Average occlusion detection rate: a ratio of the total number of correctly detected occlusion targets to the total number of occlusion targets; (4) Average direction error: the average error between the detected direction of correctly detected targets and the direction of the reference line. (The vertexes of fish head where all test sets are calibrated manually. The straight line which connects the detected position and the vertex is considered as the reference line.)

The mostly tracked trajectories (MTT), partially tracked trajectories (PTT) and times of identity switches (TIS) are used as tracking evaluation criteria [17], and their definitions are as follows: (1) Mostly tracked trajectories: the number of trajectories which are tracked for more than 80 % of ground-truth trajectories; (2) Partially tracked trajectories: the number of trajectories which are tracked between 20 % and 80 % of ground-truth trajectories; (3) Times of identity switches: the times of the identity exchange of the targets in the tracking trajectories.

### Parameter setting

There are four threshold values to be set for detection, which are *T*_*g*_, *T*_*u*_, *T*_*l*_ and *T*_*w*_ respectively. For the threshold values *T*_*g*_ and *T*_*l*_, their value is mainly determined by the size of the fish body in the image. The more pixels the fish body includes, the larger the value is and the fewer pixels the body includes, the smaller the value is. For the threshold *T*_*u*_, the value reflects how the centerline can describe the regional structures and the larger the value is, the less details can be obtained, the weaker the ability to handle the occlusion is, and the stronger the robustness is; the smaller the value is, the more details can be obtained, the stronger the ability to handle the occlusion is and the weaker the robustness is. For the threshold *T*_*w*_, its value is determined according to the average value of the width between the head and tail endpoints in the unblocked image.

Five parameters need to be set for tracking, which are *ω*, *pc*_max_, *dc*_max,_*T*_*o*_ and *N*. As for *ω*, its value is determined according to the influence of the position and direction change rate of the fish head between consecutive frames on fish movements. As for *pc*_max_ and *dc*_max_, their values are determined according to the max change of the position and direction of fish head between consecutive frames. As for *T*_*o*_, its value is determined according to the maximum occlusion distance of the target in images. The longer the distance is, the bigger its value will be, and the smaller on the contrary. As for *N*, its value is equal to the number of fish in each group.

In order to set the detection and tracking parameters, we select 500 images respectively from two test sets as training samples, analyze and compare the results obtained under different parameters with the ground-truth generated by human visual examination, and choose the parameter values with the best detection and tracking performance as setting values. The final results of parameter setting are shown in Table 1.

**Table 1:** Parameter settings in the test process

Group	Detection parameter				Tracking parameter				
	*T*_*g*_	*T*_*u*_	*T*_*l*_	*T*_*w*_	*ω*	*pc*_max_	*dc*_max_	*T*_*o*_	*N*
D1 (20 fish)	75	70	9	16	0.5	60	180	300	20
D2 (40 fish)	40	40	7	9	0.4	80	180	350	40

### Performance comparison

**Fig. 8:**
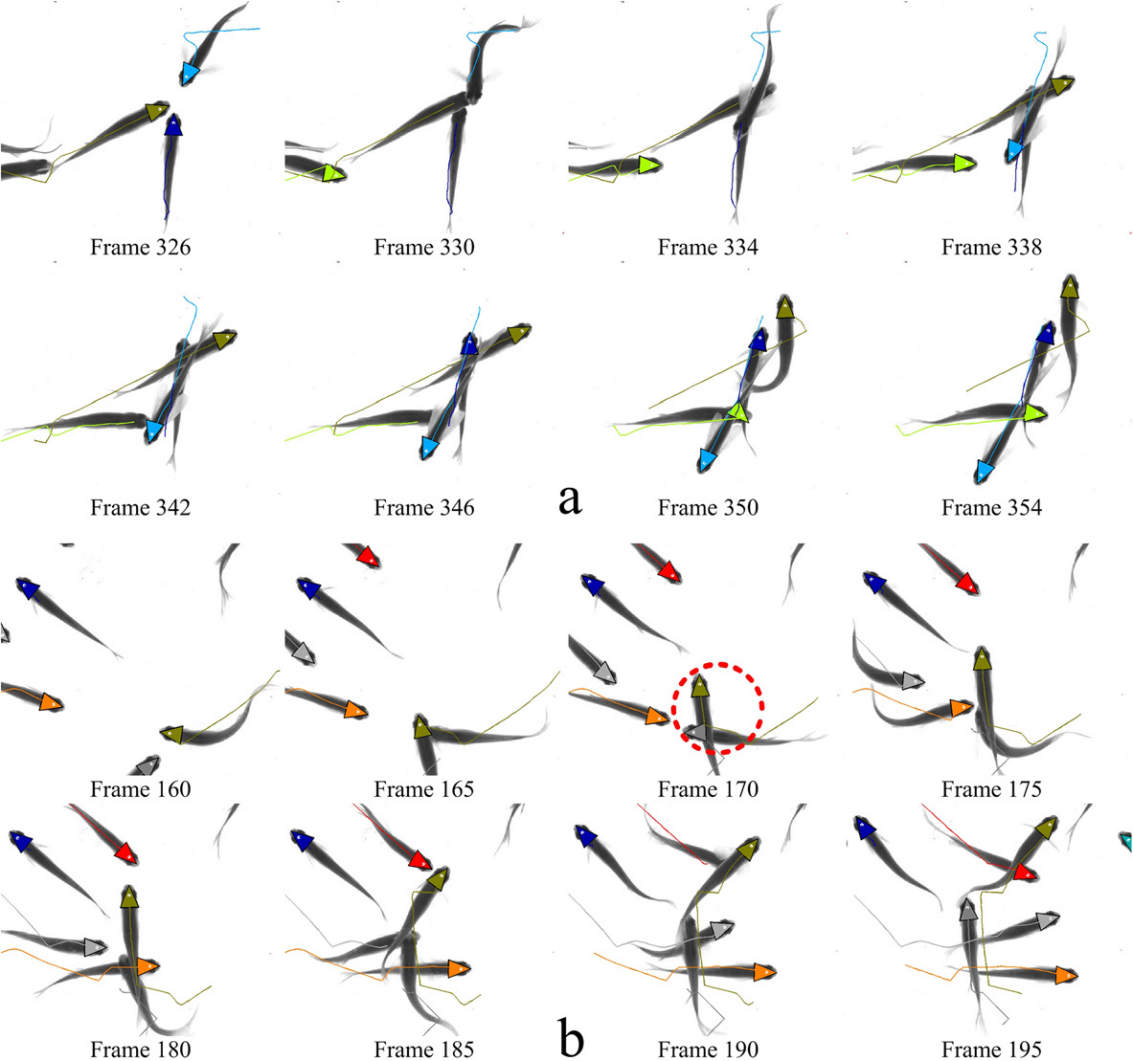
Two examples of occlusion events. **a** An occlusion event of four fish is successfully solved. **b** A case where the identity switches occurs in the tracking trajectories (the red dotted circle)

The detection results are shown in Table 2. It can be seen from the table that under different circumstances, the correct detection rate of the proposed method is above 97.1 %, and that the average direction error is less than 8.5°. This fully demonstrates the validity and accuracy of the proposed method. It is particularly worth mentioning that the error detection rate of two groups is less than 0.0002, which shows that the method is of high reliability. Moreover, the fish occlusion is relatively frequent in the two experiments, but the occlusion detection rate in the proposed method always remains above 0.795, which proves that the proposed method can deal with the fish occlusion well. Some examples of occlusion events are showed in Fig. 8. In contrast to the detection method in [18], although the occlusion detection rate in the proposed method is decreased to a certain degree, the error detection rate is also decreased, which is helpful to improve the stability of fish tracking.

**Table 2:** Detection performance on different groups

Method	Group	ACDR	AEDR	AODR	Number of occlusions	ADE
Proposed	D1 (20 fish)	0.982	0.0001	7.6	4308	0.838
	D2 (40 fish)	0.971	0.0002	8.5	10979	0.795
Blob detection and filtering [18]	D1 (20 fish)	0.986	0.009	9.4	4308	0.869
	D2 (40 fish)	0.974	0.026	10.8	10979	0.837

In order to better demonstrate tracking performance of the proposed method, it is compared with the tracking method in [18], the nearest neighbor association method [19], and the method idTracker in [11]. In the case of many targets (over 20), the global feature matching strategy of idTracker may cause a collapse of the tracking system. To improve system reliability, we replace it with local feature matching (the matching radius is *T*_*o*_). Results are given in Table 3. This table shows that the proposed method outperforms the method in [18] and the nearest neighbor association method in the two groups of tests.

**Table 3:** Tracking performance on different groups (GT: ground truth)

Method	Group	GT	MTT	PTT	TIS
Proposed	D1 (20 fish)	20	14	6	7
	D2 (40 fish)	40	32	8	9
Prediction and matching [18]	D1 (20 fish)	20	11	9	12
	D2 (40 fish)	40	26	14	17
Nearest neighbor association [19]	D1 (20 fish)	20	3	7	46
	D2 (40 fish)	40	5	16	79
idTracker [11]	D1 (20 fish)	20	18	2	2
	D2 (40 fish)	40	29	11	15

In the nearest neighbor association method, as the direction information of fish is not considered, its ability to deal with occlusion is weak and many trajectory fragments occur. Furthermore, there is also a huge increase in the times of identity switches, which greatly degrades its tracking performance.

In the method in [18], as the error detection rate is relatively high and in order to reduce the influence of error detection on the tracking performance, the strategy to combine motion prediction with feature matching is used for tracking. However, this not only increases the complexity of tracking, but also may have the problem of mismatching. While in the proposed method, as the error detection rate is relatively low, the position and direction information of targets can be directly used to conduct data association, which not only simplifies the process of tracking, but also improves the tracking performance. Thus, it can be seen that as long as the detection performance can be ensured and the direction information of fish movements can be gotten, the strategy of direct tracking without motion prediction is also feasible and effective.

IdTracker performs better than the proposed method in D1, especially when there are only two identity switches, which shows the excellent tracking performance of idTracker. In D2, the increased number of fish adds to the difficulty of identity recognition. In this scenario, idTracker degrades, while the proposed method performs better and its performance changes slightly compared with idTracker. Therefore, the proposed method can still track targets effectively even when there are more tracked targets.

**Fig. 9:**
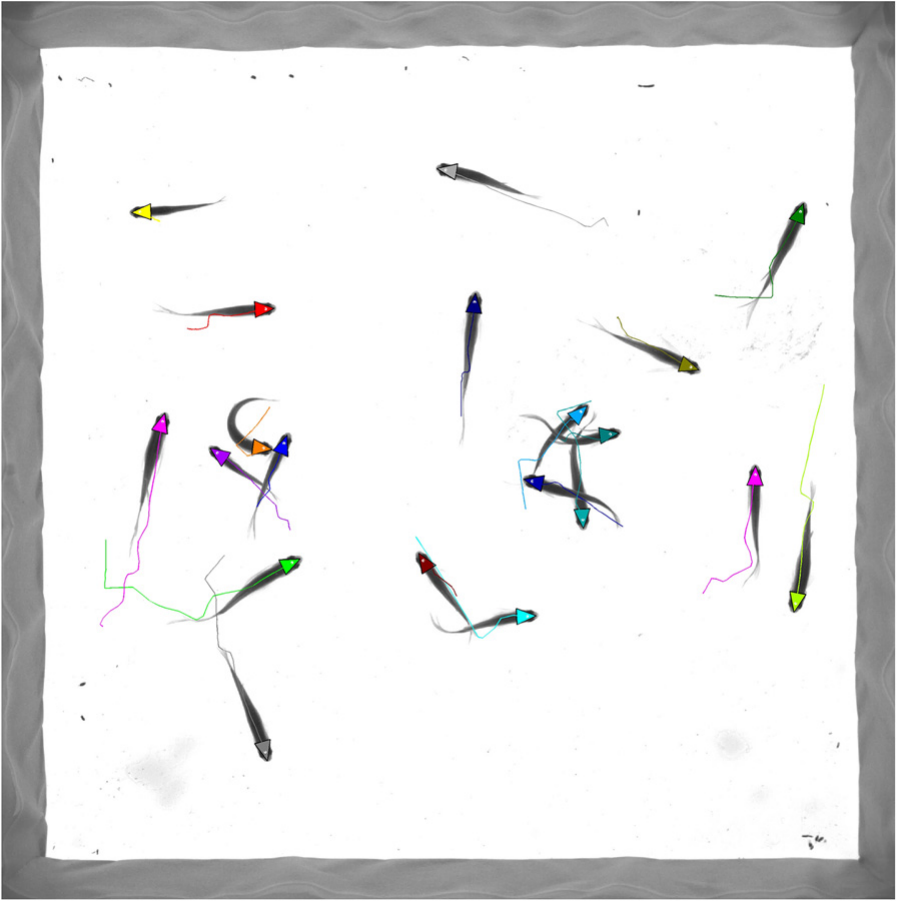
Example of frame image showing the tracking process for a group of 20 fish. The triangles denote the estimated direction of fish head, and the white points inside the triangles represent the detected position

Figure 9 shows a frame of the tracking process. In order to better observe the tracking process, we provide two additional movie files to show this in more detail (see Additional file 1 and Additional file 2).

## Conclusion

A video image fish tracking method based on fish head detection is proposed in this paper. Its contributions can be summarized as follows: (1) It takes full advantage of the most significant appearance characteristic of fish in images, that is, it analyzes fish head, and the position and direction information of fish head can be detected accurately and reliably; (2) According to the position and direction information of fish head and without motion prediction, the data association can be done directly for targets through the use of global optimization method. The experimental results show that the proposed method has a good tracking performance. Moreover, it can be seen from the experimental results that although fish movements are random, there are still some rules between the position and direction for the same target in adjacent images. A simple relationship is obtained by using only a small amount of data in this paper. Next, we will reveal these rules through more experiments.

## Abbreviations

ACDR, average correct detection rate; ADE, average direction error; AEDR, average error detection rate; AFMM, augmented fast marching method; AODR, average occlusion detection rate; DoH, Determinant of Hessian; GT, ground truth; IACUC, Institutional Animal Care and Use Committee; MTT, mostly tracked trajectories; PTT, partially tracked trajectories; TIS, times of identity switches

## Additional files

Additional file 1: Tracking result’s demo video of 20 fish. (MOV 9144 kb) Additional file 2: Tracking result’s demo video of 40 fish. (MOV 8968 kb)
